# Binary classification of non-specific low back pain condition based on the combination of B-mode ultrasound and shear wave elastography at multiple sites

**DOI:** 10.3389/fphys.2023.1176299

**Published:** 2023-04-28

**Authors:** Xiaocheng Yu, Xiaohua Xu, Qinghua Huang, Guowen Zhu, Faying Xu, Zhenhua Liu, Lin Su, Haiping Zheng, Chen Zhou, Qiuming Chen, Fen Gao, Mengting Lin, Shuai Yang, Mou-Hsun Chiang, Yongjin Zhou

**Affiliations:** ^1^ School of Biomedical Engineering, Health Science Center, Shenzhen University, Shenzhen, China; ^2^ Marshall Laboratory of Biomedical Engineering, Shenzhen, China; ^3^ Department of Medical Imaging (DMI) - Ultrasound Division, The University of Hong Kong-Shenzhen Hospital, Shenzhen, China; ^4^ School of Artificial Intelligence, OPtics and ElectroNics (iOPEN), Northwestern Polytechnical University, Xi’an, Shaanxi, China; ^5^ Department of Chinese Medicine (DCM), The University of Hong Kong-Shenzhen Hospital, Shenzhen, China; ^6^ Shenzhen Mindray Bio-Medical Electronics Co., Ltd, Shenzhen, China

**Keywords:** non-specific low back pain, ultrasound, shear wave elastography, machine learning, automatic classification

## Abstract

**Introduction:** Low back pain (LBP) is a prevalent and complex condition that poses significant medical, social, and economic burdens worldwide. The accurate and timely assessment and diagnosis of LBP, particularly non-specific LBP (NSLBP), are crucial to developing effective interventions and treatments for LBP patients. In this study, we aimed to investigate the potential of combining B-mode ultrasound image features with shear wave elastography (SWE) features to improve the classification of NSLBP patients.

**Methods:** We recruited 52 subjects with NSLBP from the University of Hong Kong-Shenzhen Hospital and collected B-mode ultrasound images and SWE data from multiple sites. The Visual Analogue Scale (VAS) was used as the ground truth to classify NSLBP patients. We extracted and selected features from the data and employed a support vector machine (SVM) model to classify NSLBP patients. The performance of the SVM model was evaluated using five-fold cross-validation and the accuracy, precision, and sensitivity were calculated.

**Results:** We obtained an optimal feature set of 48 features, among which the SWE elasticity feature had the most significant contribution to the classification task. The SVM model achieved an accuracy, precision, and sensitivity of 0.85, 0.89, and 0.86, respectively, which were higher than the previously reported values of MRI.

**Discussion:** In this study, we aimed to investigate the potential of combining B-mode ultrasound image features with shear wave elastography (SWE) features to improve the classification of non-specific low back pain (NSLBP) patients. Our results showed that combining B-mode ultrasound image features with SWE features and employing an SVM model can improve the automatic classification of NSLBP patients. Our findings also suggest that the SWE elasticity feature is a crucial factor in classifying NSLBP patients, and the proposed method can identify the important site and position of the muscle in the NSLBP classification task.

## Introduction

Low back pain (LBP) is defined as the pain between below the gluteal folds and above the vertebral protrusion that lasts for at least 1 day with or without leg pain. LBP represents one of the most common musculoskeletal disorders (Anderson, 1986; Hoy et al., 2010), with a global prevalence of 7.8% in 2017 (Buchbinder et al., 2020). Meanwhile, the number of patients increases with the population growth and aging (Hartvigsen et al., 2018; Hurwitz et al., 2018). LBP is also an important cause of physical disability (Vos, 2016), with an increasing financial burden (Woolf and Pfleger, 2003; Hoy et al., 2010; Hartvigsen et al., 2018; Hurwitz et al., 2018). LBP can be acute, with a duration of less than 6 weeks, or chronic, which lasts more than 3 months (Koes et al., 2006). Non-specific low back pain (NSLBP) refers to LBP without specific physiological or pathological causes, and its prevalence is 23%, which accounts for 90% of all LBP patients (Cheung et al., 2020). Currently, the diagnosis of LBP mainly focuses on NSLBP. Accurate and timely assessment and diagnosis of NSLBP can help the clinicians to develop effective interventions and treatments for the affected patients (Fritz et al., 2003; Liew et al., 2020).

The existing LBP prediction and assessment methods mainly focus on the subjective report of patients, biological signal and imaging studies. (Dunn and Croft, 2005) used a questionnaire to classify the severity of LBP in the enrolled patients. Aoki (2012) and Ogon et al. (1996) used a Visual Analogue Scale (VAS) to quantitatively assess NSLBP patients. However, these methods are limited by their subjective nature. Abdollahi et al. (2020) used kinematic data obtained from motion sensors combined with a STarT Back Screening Tool (SBST) questionnaire output to classify NSLBP patients into high risk and low-medium risk groups. Jiang et al. (2017) used surface electromyography (sEMG) combined with the support vector machine (SVM) algorithm to classify and identify NSLBP patients who responded to functional recovery rehabilitation, which helps healthcare workers improve the efficiency of NSLBP rehabilitation. However, the performance of these methods could be affected by the sources of their signals (sEMG or kinematic data), which are susceptible to interference from other human signals; this reduces the accuracy and stability of LBP classification. Computed tomography (CT) was used in the work of (Kamaz et al., 2007) to scan the paravertebral muscles of the L4-L5 level vertebrae in chronic LBP patients and healthy individuals. Their results showed that chronic LBP resulted in different degrees of atrophy of the muscles [most prominent in the multifidus muscle (MF)]. Furthermore, (Ketola et al., 2020) used T2-weighted magnetic resonance imaging (MRI) of the intervertebral disc for texture feature extraction. Then, NSLBP patients were divided into symptomatic and asymptomatic groups. However, CT and MRI are relatively inflexible for the physicians to screen LBP and expensive for the patients (Gilbert et al., 2004; Kamaz et al., 2007; Ketola et al., 2020).

Compared with CT and MRI, ultrasound is a non-invasive, low-cost and easy-to-use imaging tool. In recent years, it has been used to examine the skeletal muscle diseases (Cheung et al., 2020). Combined with the artificial intelligence (AI) technology, ultrasound has also been applied to the automatic classification of muscle states (Sun et al., 2020; Xu et al., 2020).

A number of studies have performed ultrasound on the muscles of the relevant parts of LBP patients. A review has discussed the studies on the diagnostic application of ultrasound in spinal canal stenosis and disc herniation (Todorov et al., 2018). Some results have shown that the MF is important in maintaining the stability of the spine (Hodges and Richardson, 1997), and most LBP patients have asymmetric MF (Fortin et al., 2019) and larger fat area (Chan et al., 2012). The thoracolumbar fascia (TLF) shear strain was reported to be lower in human subjects with chronic LBP (Langevin et al., 2011). LBP patients had a significantly smaller increase in transversus abdominis (TrA) thickness with isometric leg tasks compared with controls (Ferreira et al., 2004). The mobility of the erector spinae (ES) in LBP patients was also reported to be decreased in endurance tasks (Sanderson et al., 2019). In summary, LBP has complex causes, which involve multiple muscles and multiple sites. Hence, the diagnosis and prediction of LBP also need to combine more comprehensive information from multiple sites to achieve a more stable and robust prediction and diagnosis of LBP.

In addition to the B-mode ultrasound, shear wave elastography (SWE) has also been increasingly applied in LBP studies. Chan (Chan et al., 2012) found that the elasticity of the MF is different between LBP patients and normal people in upright and forward-stooping positions. Masaki et al. (2017) reported that the elasticity of the MF in LBP patients is significantly higher than that in normal people.

In summary, considering the complex etiology of NSLBP, it is particularly important to classify the severity or pain intensity of NSLBP and formulate corresponding treatment plans according to different classifications of NSLBP (Koes et al., 2010; Foster et al., 2011). However, an accurate manual quantification of NSLBP is difficult and time-consuming. Besides, it also relies on the physician’s subjective judgment. An automatic classification of NSLBP could help the physicians conduct prompt interventions and formulate treatment plans for the patients.

Based on the previous report of the multiple images feature selection (MIFS) (Sun et al., 2020) framework, this study integrated the B-mode ultrasound image feature from multiple sites with the SWE elasticity feature of NSLBP patients, and then employed SVM to classify NSLBP patients using the VAS as ground truth. Furthermore, we explored the importance ranking of related muscles in the diagnosis of NSLBP.

## Materials and methods

### Participants

B-mode ultrasound images and SWE elasticity of 52 NSLBP patients were collected from August 2020 to April 2021 from the University of Hong Kong-Shenzhen Hospital. Before data collection, VAS was used to evaluate the pain intensity of the patients. Each patient was informed of the purpose and process of the experiments and an informed consent form was collected from all the subjects. Ethical approval was authorized by the University of Hong Kong-Shenzhen Hospital ([2020]178).

The patients who had LBP with a significantly intense pain during rest and/or daily activities (according to VAS ≥ 1) that lasted for more than 3 months were included in the study. The exclusion criteria included history of spinal or lower limb fractures, spinal surgery or spinal deformities. According to the pain intensity, the patients were divided into two groups: 1) NSLBP patients with a mild pain (VAS ≤ 3) 2) NSLBP patients with a moderate-severe pain (VAS > 3). Data statistics are shown in Table 1.

**TABLE 1:** Descriptive statistics from the data.

	Mild (VAS ≤ 3)	Moderate-severe (VAS > 3)
Male	12	15
Female	12	13
Total	24	28
mean ± std		
Weight (Kg)	65.52 ± 11.52	64.38 ± 10.82
Height (m)	1.69 ± 0.10	1.69 ± 0.07
BMI	22.91 ± 2.72	22.54 ± 3.09
Age	35.96 ± 7.62	41.11 ± 10.24

Notes: There were no significant differences among the groups in terms of the weight, height, age or body mass index (BMI). Abbreviations: BMI, body mass index; std, standard deviation; Kg, kilogram; m, meter.

## Experimental design

### Data acquisition

Mindray DC-80 (Mindray, China) with an 8.5 MHz linear array ultrasonic transducer was used for data acquisition, which can collect B-mode ultrasound images and the SWE elasticity values (mean and standard deviation) of the muscles. In addition, the quality of the obtained SWE elasticity values was checked using DC-80 self-checking module for the elasticity imaging quality, to make the SWE elasticity measurement more stable.

**FIGURE 1:**
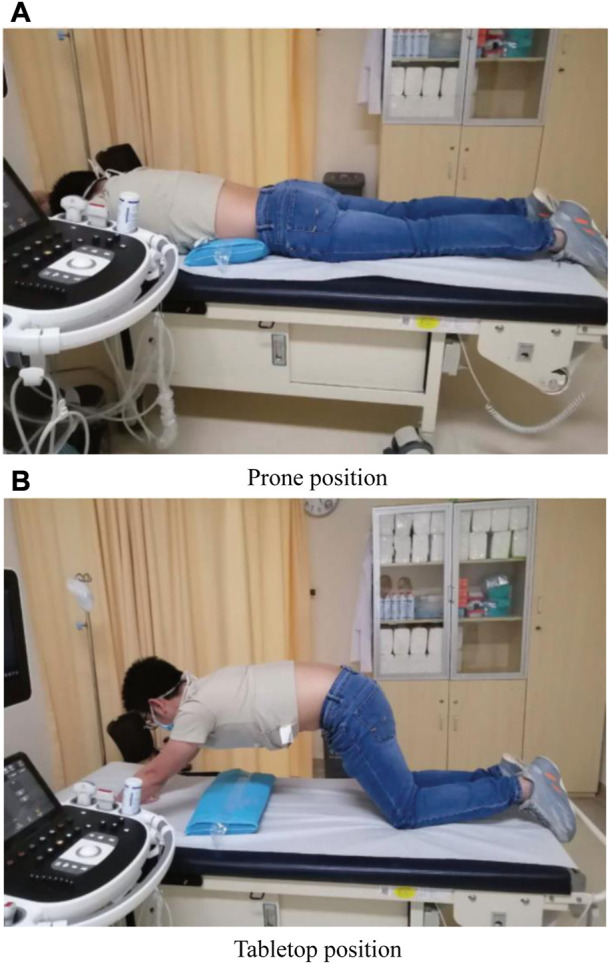
Data acquisition positions. **(A)** Prone position, **(B)** Tabletop position.

The data acquisition process included the following steps:

1) Taking the prone position, a thin pillow was put under the abdomen of the patient to make the low back flat. Then, the arms were placed flat on both sides (left side and right side) of the body, as shown in Figure 1A.
i. B-mode ultrasound images of the muscle (MF, ES, TrA and TLF) on both sides of the patient’s L2-L3 lumbar spine as well as the SWE elasticity values (mean and standard deviation) of the muscle region of interest (ROI) were acquired.
ii. B-mode ultrasound images of the MF on both sides of the patient’s L4-L5 lumbar spine as well as the SWE elasticity values (mean and standard deviation) of the muscle ROI were acquired.
2) Taking the tabletop position (Mangum et al., 2016), the patient’s low back was kept flat and relaxed, as shown in Figure 1B.
i. B-mode ultrasound images of the MF on both sides of the patient’s L2-L3 and L4-L5 lumbar spine and the SWE elasticity values (mean and standard deviation) of the muscle ROI were acquired.

**FIGURE 2:**
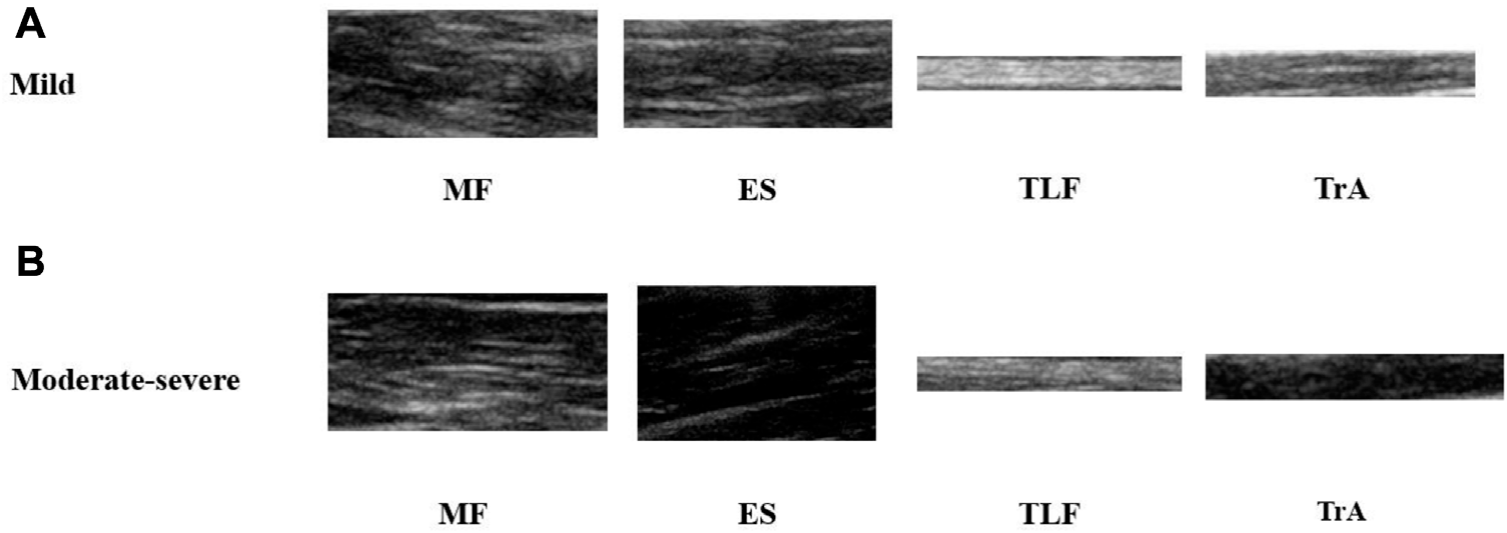
Representative images of 4 acquired muscles. **(A)** B-mode ultrasound images of NSLBP patients with a mild pain. **(B)** B-mode ultrasound images of NSLBP patients with a moderate-severe pain. MF, multifidus muscle; ES, erector spinae; TLF, thoracolumbar fascia; TrA, transversus abdominis.

Finally, we obtained the B-mode ultrasound images as well as the SWE elasticity values of a total of 14 images from the patient’s 4 muscles (MF, ES, TLF and TrA), as shown in Table 2. The representative B-mode ultrasound images are shown in Figure 2.

**TABLE 2:** Data collection sites and the patients’ positions.

	L2-L3	L4-L5	L&R	Prone position	Tabletop position	SWE elasticity
MF	**√**	**√**	**√**	**√**	√	√
ES	**√**		**√**	**√**		√
TLF	**√**		**√**	**√**		√
TrA	**√**		**√**	**√**		√

Notes: The**√** represents the imaging of the muscle in the corresponding position. Abbreviations: L2-L3, L2-L3 lumbar spine muscle; L4-L5, L4-L5 lumbar spine muscle; L&R, left and right sides of the lumbar spine muscle; SWE, shear wave elastography; MF, multifidus muscle; ES, erector spinae; TLF, thoracolumbar fascia; TrA, transversus abdominis.

### LBP framework

**FIGURE 3:**
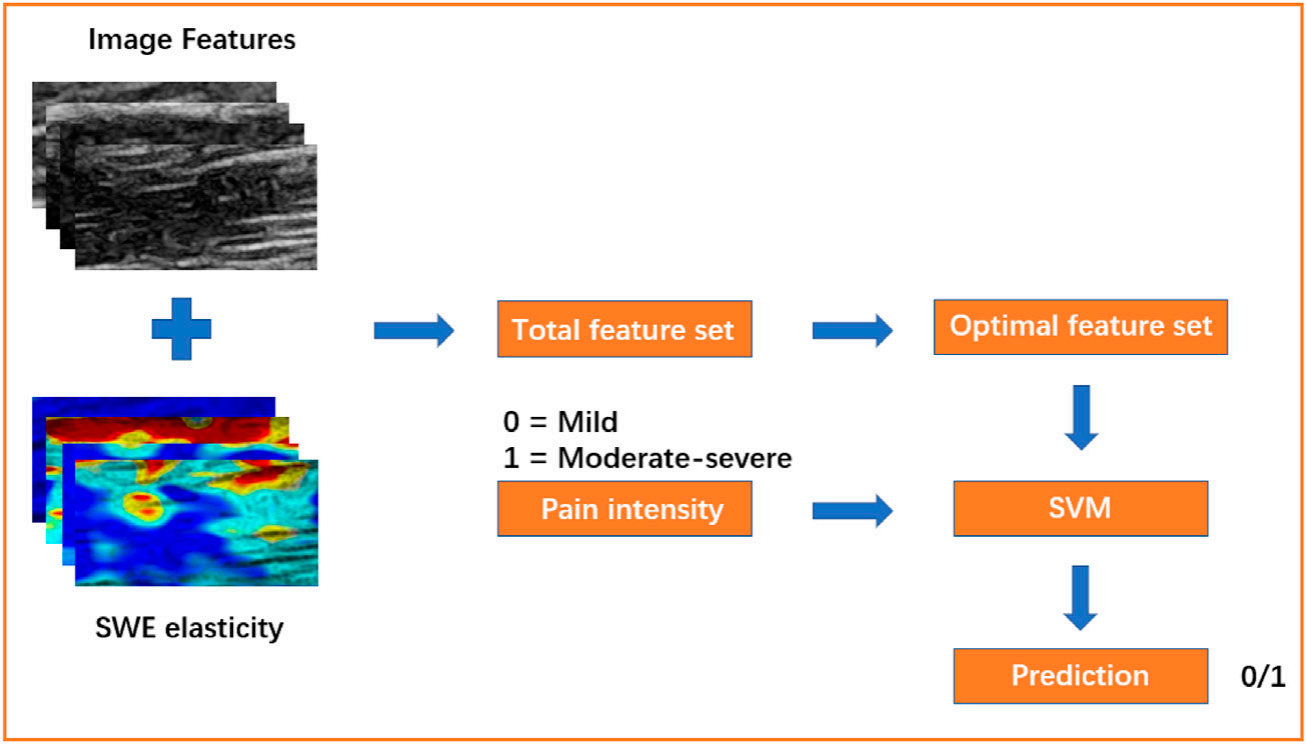
The framework of this experiment. Mild, NSLBP patients with a mild pain (VAS ≤ 3); Moderate-severe, NSLBP patients with a moderate-severe pain (VAS > 3); SVM, support vector machine.

This experiment was based on the MIFS framework, as shown in Figure 3, which was mainly composed of feature extraction and feature selection. First, 55 B-mode ultrasound image features were extracted from the ROI of muscles in multiple sites (details are shown below). Then, they were combined with the SWE elasticity feature from the ROI to form the total feature set. After feature standardization and selection, important features were selected from the total feature set to construct the optimal feature set. Finally, the optimal feature set was used to train the SVM model and classify NSLBP patients.

## Data processing

### Feature extraction

Based on the MIFS framework, this experiment extracted the features from the B-mode images as well as SWE elasticity values, including the following features:

1) Muscle morphological feature: composed of average thickness feature of the muscle (TLF and TrA). Thickness is usually defined as the distance between the midpoints of the upper and lower muscle and calculated by the mean of the left and right sides of the muscle (TLF and TrA) thickness. In order to ensure the accuracy and validity of the value, we double checked the thickness measurement.
2) Mean image frequency analysis features (MFAF) of the ROI: calculated separately as the maximum entropy method and multi-window method (Cakrak and Loughlin, 1999; Nishihara et al., 2014).
3) Image texture features: composed of two features. One is the first-order statistical (FOS) feature derived from the gray-level histogram, including the integrated optical density, mean, standard deviation, variance, skewness, kurtosis and energy. The other is the high-order texture feature, including the Haralick feature calculated from the Gray-Level Co-occurrence Matrix (GLCM) (Harlick et al., 1973), Galloway feature calculated from the Gray-Level Run-Length Matrix (GLRLM) (Galloway, 1975) as well as local binary patterns feature (energy and entropy) (Sun et al., 2020).
4) SWE elasticity feature: composed of the mean and standard deviation of SWE elasticity from the muscle ROI.

Finally, we obtained 800 features (57 features per ROI of an image and 14 images per subject) and 2 average thickness features of the TLF and TrA of each subject. The details of the features are listed in Table 3.

**TABLE 3:** Details of the features.

Feature type	Feature names	Notes
Muscle morphological feature	muscle (TLF and TrA) average thickness feature	N = 2
Image frequency analysis feature	MFAF	Calculated by the maximum entropy method and multi-window method. N = 2
FOS feature	Integrated optical density, mean, standard deviation, variance, skewness, kurtosis and energy	Derived from the gray-level histogram. N = 7
Haralick feature	Contrast, correlation, energy, entropy, homogeneity and symmetry	Calculated from the Gray-Level Co-occurrence Matrix (GLCM) with 4 directions: 0°, 45°, 90° and 135°. N = 24
Galloway feature	Short Run Emphasis (SRE), Long Run Emphasis (LRE), Gray-Level Non-Uniformity (GLNU),	Calculated from the Gray-Level Run-Length Matrix (GLRLM) with 4 directions: 0°, 45°, 90°, and 135°. N = 20
	Run Length Non-Uniformity (RLNU) and Run Percentage (RP)	
Local binary patterns feature	Energy and entropy	N = 2
SWE elasticity feature	Mean and std	N = 2

Abbreviations: N, the number of features; TLF, thoracolumbar fascia; TrA, transversus abdominis; FOS, first-order statistical; MFAF, mean image frequency analysis feature; SWE, shear wave elastography; std, standard deviation.

### Feature selection

1) The variance between the features of the total feature set obtained in this experiment was relatively large. To prevent the variance of some features from being much larger than the variance of other features, which could result in slow convergence or non-convergence of the model, this experiment standardized the total feature set to make the mean and variance of the feature equal to 0 and 1, respectively, as shown in Eq. 1.

$$y=\frac{x-\mu }{\sigma }$$ (1)

where y is the feature after standardization, x is the feature before standardization, μ is the mean of the feature set, and σ is the standard deviation of the feature set.

2) In this experiment, the number of features obtained by each patient was 800. To prevent the model from overfitting or having difficulty converging, it was necessary to perform feature selection on the total feature set and to reduce the feature dimension. We used *SelectPercentile* of *sklearn.feature_selection* in the machine learning package *Scikit-learn (v. 0.22.1)* for feature selection. The *score_func* parameter contains several feature selection methods. In this experiment *score_func* chose the default *f_classif* as the feature selection method, and the kernel method of *f_classif* was the analysis of variance (ANOVA) (Larson, 2008). Due to the ability to combine all the feature information, ANOVA does not only improve the efficiency, but also increases the reliability of the feature selection. After feature selection with ANOVA, the optimal feature set was finally extracted from the total feature set.

### Classification

1) The *Scikit-learn (v. 0.22.1)* machine learning library was used in *Python (v. 3.7.6)* to build a machine learning-based pipeline to analyze the feature data.
2) SVM was used to classify NSLBP patients. SVM is a two-classification model, and its basic model is a linear classifier with the largest interval defined in the feature space. The basic idea behind SVM is to solve the separation hyperplane that can correctly divide the dataset and have the largest geometric interval. SVM has several complex kernel methods, which can make the data that are inseparable in the linear space separable in other dimensions. At the same time, the addition of regularization enhances the robustness of the SVM model and reduces the possibility of overfitting. The SVM model of this experiment used a linear kernel and L2 regularization.
3) When the amount of data is sufficient, datasets are usually divided into training set, validation set and test set, and the final performance results come from the test set. However, since the amount of data in this experiment is limited, the above-mentioned single test set could not reliably report the performance indicators of the classifier (Sun et al., 2020). To overcome this disadvantage, five-fold cross-validation combined with grid search was used to optimize and evaluate the classification model in this experiment.
4) In the classification task of NSLBP of this experiment, NSLBP patients with a moderate-severe pain were treated as positive cases and those with a mild pain as negative ones. The metrics of accuracy, specificity, sensitivity, AUC (area under the receiver operator characteristic curve), precision and negative predictive value (NPV) were used to quantify the classification results. At the same time, the classification results of using the SWE elasticity feature, B-mode ultrasound image feature and SWE elasticity feature combined with B-mode ultrasound image feature were compared.

## Results

In this section, the results of feature selection using ANOVA and SVM to classify NSLBP are presented. At the same time, this section also exhibits the number of specific features in the optimal feature set after feature selection, the top 10 important features of the SVM model, the proportion of 4 muscles (MF, TLF, ES, TrA) in the optimal feature set, the proportion of different positions and sites in the optimal feature set, as well as the classification results between MIFS framework and single image feature selection (SIFS) framework.

### Feature analysis

**FIGURE 4:**
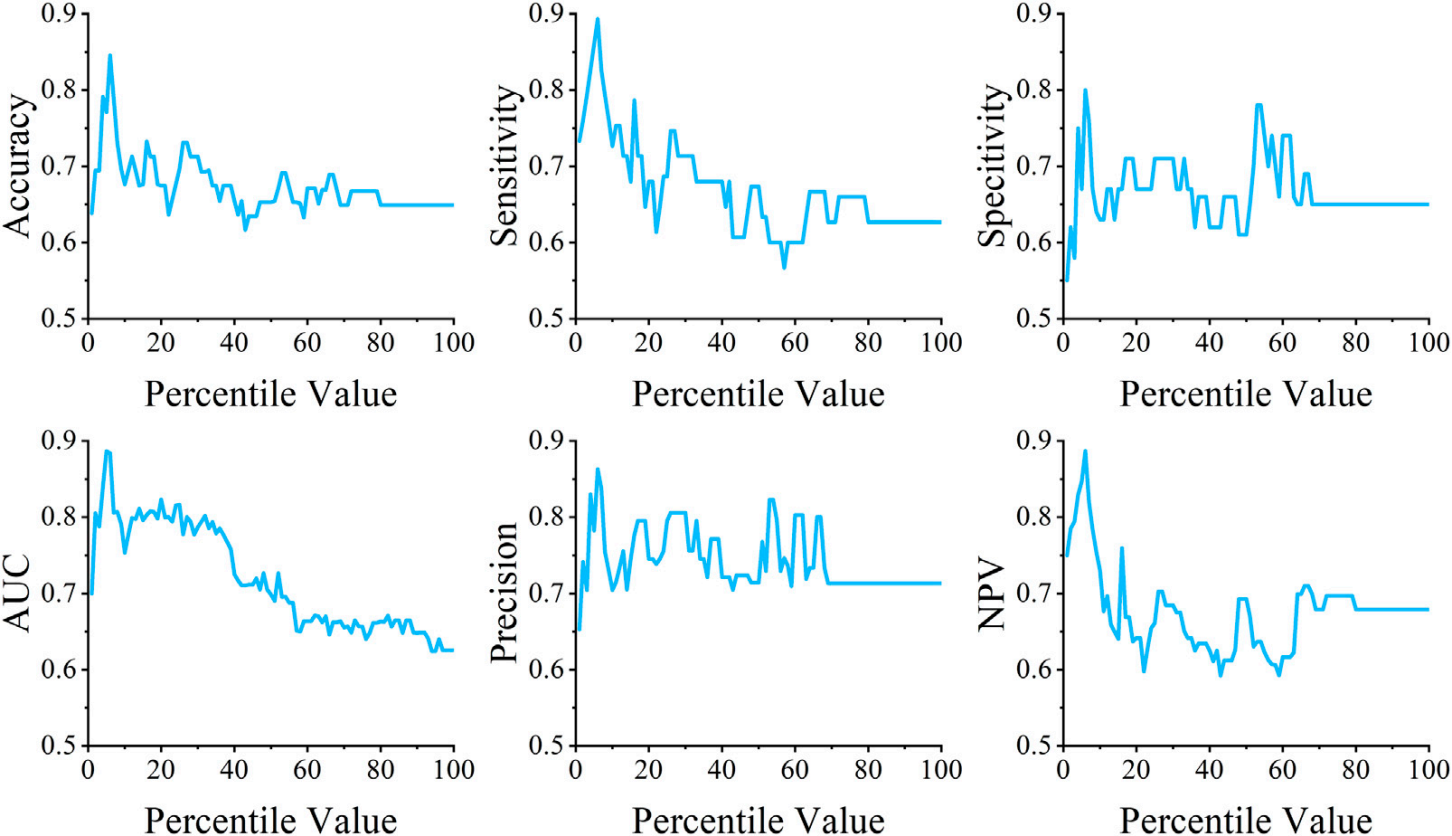
The performance of feature selection with different values of the *percentile* parameter.

In order to find out the best performance in different parameters, the *percentile* parameter of *SelectPercentile* was set from 1 to 100 for feature selection. When the *percentile* was set to 6, the model achieved the best performance, as shown in Figure 4

After feature selection, 48 features were finally obtained in the optimal feature set, which accounted for 6% of the total feature set. Table 4 shows the number of different features in the optimal feature set.

**TABLE 4:** Specific number of features in the optimal feature set.

Feature type	Muscle morphological feature	Image frequency analysis feature	FOS feature	Haralick feature	Galloway feature	Local binary patterns feature	SWE elasticity feature	Total
Select No.	0	2	3	27	12	0	4	48
Total No.	2	28	98	336	280	28	28	800

Abbreviations: FOS, first-order statistical; SWE, shear wave elastography.

### The classification performance

Table 5 shows the classification results of different feature sets after performing five-fold cross-validation using SVM. It can be observed that the accuracy of using the SWE elasticity feature and B-mode ultrasound image feature to classify NSLBP was 0.65 and 0.81, respectively. When both were used to classify NSLBP patients, the accuracy reached 0.85.

**TABLE 5:** Classification results.

Feature set	SWE elasticity	B-mode ultrasound image feature	Total
Accuracy	0.65	0.81	0.85
Sensitivity	0.76	0.82	0.89
Specificity	0.54	0.80	0.80
AUC	0.66	0.80	0.88
Precision	0.69	0.86	0.86
NPV	0.70	0.82	0.89

Abbreviations: AUC, area under curve; NPV, negative predictive value; SWE, shear wave elastography.

In the SVM model, the absolute value of the feature weight can indicate the importance of the feature in the classification task. In this experiment, the top 10 important features obtained in the SVM model are shown in Table 6. We conducted the Student’s *t*-test and Mann-Whitney test in the top 10 important features came from the normal distribution or not, respectively. As shown in Table 6 (indicated by asterisks), all the 10 features showed statistically significant differences.

**TABLE 6:** Top 10 important features in the optimal feature set.

L4-L5_L_MF_FisrtOrderFeature.IOD (prone position) *
L2-L3_R_MF_SWE.Std (prone position) *
L2-L3_R_TLF_GallowayFeature.RLNU (prone position, direction = 0°) *
L2-L3_R_TLF_HaralickFeature.Homogeneity (prone position, direction = 90°) **
L2-L3_L_MF_GallowayFeature.RLNU (tabletop position, direction = 90°) **
L4-L5_R_MF_SWE.Std (tabletop position) **
L2-L3_L_ES_SWE.Std (prone position) *
L2-L3_R_TLF_MFAF (prone position, calculated by multi-window method) *
L2-L3_L_MF_GallowayFeature.GLNU (tabletop position, direction = 0°) **
L2-L3_L_MF_GallowayFeature.RP (tabletop position, direction = 0°) *

Abbreviations: L2-L3, L2-L3 lumbar spine muscle; L4-L5, L4-L5 lumbar spine muscle; L, left side of the lumbar spine muscle; R, right side of the lumbar spine muscle; SWE, shear wave elastography; Std, standard deviation; GLNU, gray-level non-uniformity; MF, multifidus muscle; ES, erector spinae; TLF, thoracolumbar fascia; TrA, transversus abdominis; *, *p*-value < 0.05; **, *p*-value < 0.01.

## Discussion

1) Performance comparison between the proposed method and the MRI work

This experiment combined the B-mode ultrasound image feature with the SWE elasticity feature to classify NSLBP patients. Compared with the MRI work in NSLBP classification (Ketola et al., 2020), the accuracy and sensitivity after five-fold cross-validation of this experiment were 0.85 and 0.89, respectively, which were higher than MRI work (0.83 and 0.82, respectively), suggesting that the proposed method can better identify NSLBP patients with a moderate-severe pain. The precision was 0.86 in this experiment, which is much higher than the MRI work (0.56), indicating that the classification of the proposed method is more robust in classifying positives than the MRI work. NPV refers to the proportion of true negatives among all negative results, the NPV and specificity of this experiment were 0.89 and 0.80, respectively, lower than those of the MRI work (0.94 and 0.83, respectively), which means a relatively low proportion of classifying negatives and indicates that NSLBP patients with a mild pain are relatively more difficult to be identified in this experiment. The AUC of this experiment was 0.88, lower than MRI work (0.91), which suggests that the MRI work may be more robust in NSLBP classification. We understand that direct comparisons between different studies can be challenging due to variations in patient populations, imaging techniques, and other factors. In future work, we plan to validate our approach on larger and more diverse datasets, including comparisons with other imaging modalities, such as MRI.

It should also be noted that the NSLBP classification in this experiment is based on the pain intensity evaluated by VAS, which is a subjective variable. However, all the patients have experienced significant pain for more than 3 months, and we obtained a relatively low proportion of false positives in the classification results, which is desirable in medical research.

2) Involving SWE elasticity makes a difference

The classification results indicate that using the SWE elasticity feature or using B-mode ultrasound image feature for classification is not as good as combining both in the classification of NSLBP patients. Besides, the classification results also confirmed the importance of SWE in the clinical research (Zhou et al., 2018).

3) A brief discussion from the perspective of features

In this study, an optimal feature set with the size of 48 was obtained after feature selection (as shown in Table 4). Among the total feature proportion in the optimal feature set, the proportion of the SWE elasticity feature was the highest, achieved 14.3% (4/28), which indicates that SWE features plays an important role in the NSLBP classification in this experiment. This also preliminarily confirms the previous experiments by Chan et al. (2012) and Masaki et al. (2017) (Fortin et al., 2019; Sanderson et al., 2019) that the SWE elasticity of LBP patients is different from that of normal people and the SWE elasticity of LBP patients could change.

By analyzing the top 10 important feature weights of the SVM classification model, the Galloway feature accounted for 40.0% of the entire classification task (as shown in Table 6), indicating that the Galloway feature calculated by the GLRLM could well show the difference in the B-mode ultrasound image features between NSLBP patients.

4) A brief discussion from the perspective of muscles

As shown in Table 7, among 48 selected features from 800 total features, the features of MF and TLF had the highest proportions, reaching 4.4% (20/456) and 22.6% (26/115), respectively, while ES and TrA had the lowest proportions, reaching 1.6% (2/114) and 0.0% (0/115), respectively. It can be seen that MF and TLF play an important role in the classification of NSLBP patients (Kamaz et al., 2007; Chan et al., 2012), which indicates that more attention should be paid to the MF and TLF in NSLBP classification in future experiments.

5) A brief discussion from the perspective of positions and sites

**TABLE 7:** The number of features from different muscles in the optimal feature set.

Muscle	MF	ES	TLF	TrA	Total
Select No.	20	2	26	0	48
Total No.	456	114	115	115	800

Abbreviations: MF, multifidus muscle; ES, erector spinae; TLF, thoracolumbar fascia; TrA, transversus abdominis.

As shown in Table 8, the feature of the L4-L5 lumbar spine muscle accounted for 0.9% (2/228) in 48 selected features from 800 total features, while the feature of L2-L3 lumbar spine muscle accounted for 8.0% (46/572), indicating that the L2-L3 lumbar spine muscle was more important than the L4-L5 lumbar spine muscle in the classification of NSLBP in this experiment. The feature proportion of the left side of the lumbar spine muscle was 5.5% (22/399), while the right side of the lumbar spine muscle was 6.5% (26/399), which indicates that both sides of the lumbar spine muscle contributed equally to the NSLBP classification in this experiment in some extent. As for the data acquisition position, the tabletop position had more contribution [accounted for 6.1% (14/228)] than the prone position [accounted for 5.9% (34/572)], which verifies the importance of the tabletop position in the imaging of related muscles (Foster et al., 2011).

**TABLE 8:** The number of features at different positions and sites of the optimal feature set.

Positions and sites	L2-L3	L4-L5	L	R	Prone position	Tabletop position
Select No.	46	2	22	26	34	14
Total No.	572	228	399	399	572	228

Abbreviations: L2-L3, L2-L3 lumbar spine muscle; L4-L5, L4-L5 lumbar spine muscle; L, left side of the lumbar spine muscle; R, right side of the lumbar spine muscle.

In this experiment, the MIFS framework extracts features from all 14 images in multiple sites and searches for the best feature set. As shown in Table 9 **,** compared with the SIFS framework extracts the feature from a single image in single site, the accuracy, specificity, AUC, precision and NPV of MIFS framework were higher than SIFS framework, which indicated that MIFS framework had a better performance in NSLBP classification.

**TABLE 9:** MIFS framework and SIFS of NSLBP classification performance.

Muscle sites	Accuracy	Sensitivity	Specificity	AUC	Precision	NPV
L2-L3	0.67	0.93	0.38	0.61	0.64	0.87
_L_MF						
L2-L3	0.63	0.93	0.30	0.61	0.62	0.65
_R_MF						
L4-L5	0.68	0.65	0.71	0.66	0.71	0.66
_L_MF						
L4-L5	0.69	0.80	0.59	0.72	0.73	0.78
_R_MF						
L2-L3_L	0.77	0.75	0.79	0.79	0.81	0.76
_MF_TBT						
L2-L3_R	0.75	0.83	0.65	0.75	0.75	0.77
_MF_TBT						
L4-L5_L	0.67	0.67	0.66	0.69	0.76	0.67
_MF_TBT						
L4-L5_R	0.77	0.89	0.62	0.74	0.76	0.88
_MF_TBT						
L2-L3	0.66	0.72	0.58	0.61	0.71	0.62
_L_ES						
L2-L3	0.65	0.85	0.41	0.61	0.65	0.65
_R_ES						
L2-L3	0.73	0.75	0.70	0.78	0.77	0.71
_L_TLF						
L2-L3	0.75	0.89	0.58	0.77	0.71	0.83
_R_TLF						
L2-L3	0.62	0.67	0.54	0.61	0.69	0.59
_L_TrA						
L2-L3	0.71	0.68	0.75	0.68	0.78	0.67
_R_TrA						
Total	0.85	0.89	0.80	0.88	0.86	0.89

Abbreviations: L2-L3, L2-L3 lumbar spine muscle; L4-L5, L4-L5 lumbar spine muscle; L, left side of the lumbar spine muscle; R, right side of the lumbar spine muscle; TBT, tabletop position; MF, multifidus muscle; ES, erector spinae; TLF, thoracolumbar fascia; TrA, transversus abdominis; AUC, area under curve; NPV, negative predictive value; MIFS, multiple images feature selection; SIFS, single image feature selection.

In this experiment, the data acquisition repeatability test was performed on the SWE elasticity values (Yu, 2022). The SWE elasticity values of 10 people were measured by two clinical operators at different times, and significant differences were found in the SWE elasticity of the TLF between the two operators, which indicated possible differences between the operators in the SWE elasticity measurement of the TLF. Furthermore, the choice of the ROI of the B-mode ultrasound image in this experiment depended on the experience of the clinical operator, and there was no fixed size. In future experiments, with the use of automatic methods, such as deep learning-based methods, the subjective influence of the operators can be reduced. At the same time, with the increase of the dataset size in feature experiments, the classification model can be more robust.

Finally, although we used the VAS method as ground truth to train our classifier, our proposed method has potential advantages over the subjective nature of VAS. While the VAS method is a widely used tool for assessing pain, it is indeed limited by its subjective nature, as the patient’s perception and reporting of pain can be influenced by various factors. However, it remains a commonly used method for assessing pain in clinical settings, including in the assessment of NSLBP. As such, we used the VAS as the ground truth in our study because it represents a standard and commonly used method for assessing pain in NSLBP patients. The significance of our study lies in the development of an automated method for classifying NSLBP patients using B-mode ultrasound and SWE features, which has the potential to improve the accuracy and objectivity of NSLBP assessment. Our proposed method has several advantages over the VAS method. First, it can provide an objective measure of NSLBP severity that is not dependent on patient self-reporting. Second, our method has the potential to reduce inter-observer variability, which is a known limitation of manual quantification methods. Third, our proposed method is cost-effective and non-invasive, which may make it more accessible and practical in clinical settings. Meanwhile, Low back pain is a complex condition that can arise from various sources. It is crucial to recognize that changes in the paraspinal muscles may not necessarily be the primary cause of the pain, but rather a secondary effect of underlying factors. Therefore, it is essential to explore a wide range of possible pain generators when diagnosing low back pain. It is important to note that our study’s emphasis on the paraspinal muscles is not all-encompassing, and there may be other contributors to low back pain that require consideration.

## Conclusion

In conclusion, the current study utilized an SVM model in combination with the MIFS framework and B-mode ultrasound image feature and SWE elasticity feature to classify NSLBP patients. The MIFS framework, previously proven effective in motion level classification, was employed due to the complicated etiology of LBP and the observed differences in multiple muscle sites of LBP patients compared to normal individuals. The proposed approach achieved better performance than the SIFS framework, providing preliminary evidence for the potential of integrating multiple sites of B-mode ultrasound image and SWE elasticity features in the classification of NSLBP patients.

## Data Availability

The datasets presented in this article are not readily available because of privacy concerns. Requests to access the datasets should be directed to corresponding author.
